# Strategies for the biocontrol *Pseudomonas* infections pre‐fruit harvest

**DOI:** 10.1111/1751-7915.70017

**Published:** 2024-10-04

**Authors:** Suzanne L. Warring, Hazel M. Sisson, Peter C. Fineran, Mojgan Rabiey

**Affiliations:** ^1^ Department of Microbiology and Immunology University of Otago Dunedin New Zealand; ^2^ Maurice Wilkins Centre for Molecular Biodiscovery, University of Otago Dunedin New Zealand; ^3^ Bioprotection Aotearoa, University of Otago Dunedin New Zealand; ^4^ Genetics Otago Dunedin New Zealand; ^5^ School of Life Sciences University of Warwick Coventry UK

## Abstract

The efficiency of global crop production is under threat from microbial pathogens which is likely to be worsened by climate change. Major contributors to plant disease are *Pseudomonas syringae* (*P. syringae*) pathovars which affect a variety of important crops. This opinion piece focuses on *P. syringae* pathovars *actinidiae* and *syringae*, which affect kiwifruit and stone fruits, respectively. We discuss some of the current control strategies for these pathogens and highlight recent research developments in combined biocontrol agents such as bacteriophages and combinations of bacteriophages with known anti‐microbials such as antibiotics and bacteriocins.

## Graphical abstract

Bacteriophages, phage‐derived proteins and organic acids offer potential as biocontrols for bacterial pathogens that affect fruit production. Our opinion piece outlines current developments in phage biocontrol and where the field is moving to next in the quest to create environmentally sustainable methods for pest management.

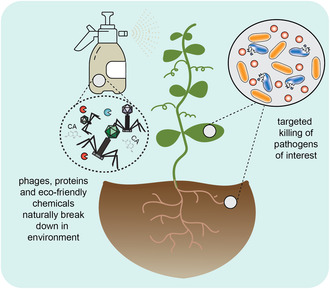

## BACKGROUND

As the global population rises, so does the requirement for increased food production. However, food production faces threats not only in the continued extreme weather events due to climate change but additional increased risk of dissemination of bacterial pathogens already implicated in crop loss and damage (Roussin‐Léveillée et al., 2024; Singh et al., 2023). Current estimates show that microbial pathogens have a ~US$220 billion annual effect on crop production (Singh et al., 2023). Severe crop damage has been caused globally by various *Pseudomonas syringae* (*P. syringae*) pathovars, with over 50 pathovars identified as causing disease in various plants species (Xin et al., 2018).

*Pseudomonas syringae* is a highly versatile and complex species of bacteria that includes several pathovars capable of infecting a wide range of economically important crops worldwide, including stone fruits such as cherries, plums, and apricots; pome fruits like apples and pears; berries such as kiwi, and vegetables such as tomatoes, peppers, and beans. The pathogen is known for causing various plant diseases, such as bacterial canker, which manifests as cankers on tree trunks and branches, leading to dieback and eventual tree death; bacterial blight, characterized by necrotic lesions and wilting on leaves, stems, and fruits; leaf spot, resulting in unsightly spots on foliage that can weaken plants and affect their overall vigour and blossom blast, which destroys blossoms and young fruit, directly impacting reproductive capacity and yield. These diseases lead to significant economic losses for farmers due to reduced crop quality and yield, as well as increased management and control costs. Infected crops may face market restrictions or trade barriers due to poor quality. The global impact of *P. syringae* underscores the need for sustainable and effective management strategies, including ongoing research into integrated pest management approaches that utilize biocontrol agents to ensure the long‐term health and productivity of crops worldwide.

The global economic damage caused by *P. syringae* in agriculture and horticulture is difficult to quantify precisely due to varying conditions such as regional climates, differing crop types, and the specific strains of the bacteria that may be present. While specific figures can vary greatly depending on the crops and regions affected, *P. syringae* poses a significant threat to agricultural and horticultural productivity and profitability, highlighting the need for continuing investigation into effective management and control strategies.

In this opinion piece we focus on the pathogens *P. syringae* pv. *actinidiae* and pv. *syringae* which affect kiwifruit and stone fruits, respectively. We address the limitations of current control strategies and highlight recent developments in research towards utilizing bacteriophages (phages) and phage combination approaches for control of these pathogens.

## *PSEUDOMONAS SYRINGAE* PV. *ACTINIDIAE* THE CAUSAL AGENT OF KIWIFRUIT CANKER

*Pseudomonas syringae* pv. *actinidiae* (*Psa*) first emerged in kiwifruit orchards in Japan in the 1980s. It subsequently spread to the majority of kiwifruit producing countries such as China, South Korea, Italy, New Zealand, Spain and parts of South America. *Psa* has been classed into 5 biovars based on genetic diversity and toxin production (Fujikawa & Sawada, 2016), with biovar 3 implicated in the most recent pandemic which emerged in 2009. Disease caused by *Psa* has resulted in severe economic damage to the global kiwifruit industry, with an estimated cost of ~$2 billion NZD to the NZ economy alone (Vanneste, 2017). At its initial emergence, the primary control measure for affected orchards was widespread removal and destruction of affected vines. More susceptible cultivars were then replaced with kiwifruit variants that have greater tolerance to *Psa* such as the gold3 and green14 cultivars (Ashrafzadeh & Leung, 2019). Currently, *Psa* is controlled using copper and antibiotic spray regimes, along with plant elicitors such as Actigard™ and more recently developed biocontrol tools such as Aureo Gold™ (Pereira et al., 2021).

## *PSEUDOMONAS SYRINGAE* PV. *SYRINGAE* IN STONE FRUIT PRODUCTION

*P. syringae* pv. *syringae* (*Pss*) is a highly versatile and destructive pathogen that poses significant threats to various economically important crops, particularly stone fruits such as cherry and plum. In New Zealand and the United Kingdom, disease in orchards is predominantly caused by *Pss*, which is classified into Phylogroup 2 (Hulin et al., 2020; Marroni et al., 2023). *Pss* can cause a range of devastating diseases, including bacterial canker, which severely diminishes fruit quality and yield. The pathogen is known for its ability to infect all parts of the tree, resulting in numerous issues including the formation of shot holes in leaves, necrotic lesions on fruits, and extensive oozing and bleeding on tree trunks. In severe infections, the damage caused by *Pss* can be so extensive that it threatens the survival of the affected trees. The loss of trees, coupled with the reduced productivity and quality of fruit, can lead to substantial economic repercussions for fruit growers. Additionally, these infections have a negative impact on timber production, as the pathogen affects the quality and quantity of timber harvests. The overall devastation caused by *Pss* in both the fruit and timber industries underscores the urgent need for effective management strategies to control this pathogen and protect valuable agricultural and forestry resources.

## THE LIMITATIONS IN CURRENT CONTROL METHODS USED TO CONTROL PHYTOPATHOGENS

**FIGURE 1:**
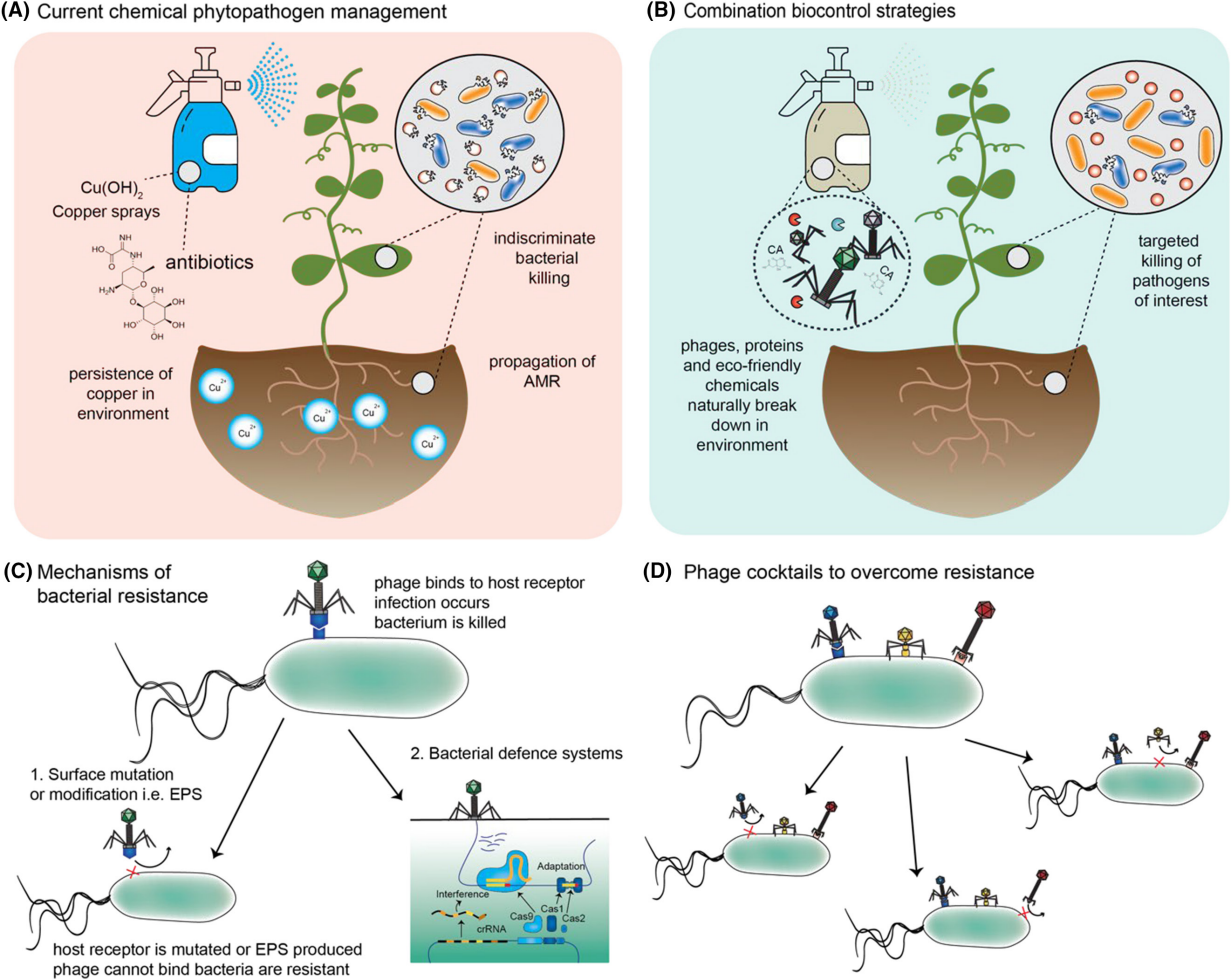
Current control strategies, proposed new biocontrol strategies and bacteriophage infection strategy and overcoming resistance. (A) Drawbacks of current methods used to control *Ps* and *Psa* in kiwifruit and stone fruit orchards, respectively. Both pathogens are controlled using copper and antibiotic sprays. (B) Advantages of using phage and combination phage biocontrols for the control of phytopathogens in food production. (C) Mechanism by which phage infect their host bacteria and how host bacteria can develop resistance. Resistance can be developed through 2 mechanisms: (1) surface mutation of the phages host receptor, and (2) host defence systems such as CRISPR‐Cas, restriction modification (RM), bacteriophage exclusion (BREX) systems, toxin/anti‐toxin etc. (Mayo‐Muñoz et al., 2023). (D) Utilization of phage cocktails using multiple phages with variable receptors and different functionalities to escape surface mutations or EPS production of phage resistant bacteria.

Chemical treatments, particularly copper‐based bactericides, have historically been the primary method for managing *P. syringae* infections (Figure 1A). These treatments were favoured for their immediate and broad‐spectrum anti‐bacterial effects. However, over time, their efficacy has proven to be inconsistent, and they can harm the environment (Pereira et al., 2021). The persistence and accumulation of copper in soil can lead to toxicity, impacting beneficial soil microorganisms, plants, and aquatic ecosystems when washed away by runoff. Additionally, the overuse of chemical treatments has contributed to the evolution of resistance in *P. syringae* populations (Colombi et al., 2017). As a result, once‐effective chemical treatments are now losing their ability to control *P. syringae* effectively. Resistance development is further exacerbated by cross‐resistance, where resistance to one type of chemical can lead to resistance against others (Sundin & Wang, 2018).

Furthermore, increased regulatory restrictions on the use of chemicals and antibiotics in agriculture are limiting their application. For instance, the EU propose (European Union: Sustainable Use of Pesticides, 2022) a 50% reduction in pesticide use by 2030. The push towards more sustainable and environmentally friendly farming practices has further reduced the availability and utility of chemical treatments. This necessitates the exploration of alternative control strategies including new biocontrol agents such as bacteriophages and commensal microbes (Baig et al., 2012; Fira et al., 2018; Roca et al., 2024; Shao et al., 2023) for inclusion in integrated pest management practices to combat *P. syringae* infections effectively and sustainably.

## BACTERIOPHAGES AND THEIR POTENTIAL AS BIOCONTROL AGENTS

Bacteriophages, or phages, are viruses that specifically infect and lyse bacteria (Hampton et al., 2020). They are the most abundant organisms on Earth, naturally present in various environments, including soil, water, and plants. Phages have gained attention as a promising biocontrol strategy against bacterial pathogens due to their specificity and capacity to quickly and effectively reduce bacterial populations (Figure 1B). Phages offer several advantages as biological control agents. Phages offer specific targeting of bacterial strains without affecting non‐target organisms, minimizing collateral damage to beneficial microbes and the surrounding ecosystem. Additionally, phages can be applied directly to plants as a spray or incorporated into formulations such as soil treatments for sustained release, providing flexibility in application methods. Phages that target both *Pss* (Holtappels et al., 2024; Rabiey et al., 2020) and Psa (Bai et al., 2022; Flores et al., 2020; Liu et al., 2021; Martino et al., 2021; Pinheiro et al., 2020; Song et al., 2021; Yin et al., 2019; Yu et al., 2015) have been isolated from various environments, such as soil, plant surfaces, and water bodies. Our recent work identified phages capable of targeting both *Pss* and *P. syringae* pv. *morsprunorum* (another causative agent of cherry canker), demonstrating their efficacy in reducing bacterial populations in both in vitro and in planta settings (Rabiey et al., 2020, 2024). Further, we have previously isolated and characterized phages against *Psa* (Frampton et al., 2014, 2015; Wojtus et al., 2017) for the purposes of phage biocontrol in kiwifruit. The development of phage cocktails is integral to enhance the effectiveness of phage biocontrols, address potential resistance issues and to cover the range of target pathogens (Wang et al., 2019). Bacterial resistance to phages can rapidly occur via two broad routes (extracellular and intracellular) (Figure 1C) and includes (1) alteration of the bacterial surface through mutation of the phage receptor or production of extra polymeric substances (EPS) which occlude the host receptor, or (2) the action of host defence systems such CRISPR‐Cas, RM, BREX systems and toxin/anti‐toxin, amongst others (Mayo‐Muñoz et al., 2023). Phage cocktails should contain multiple phages that can have different host ranges or utilize differing host receptors (Figure 1D). By using diverse phages in cocktails (Figure 1D), the host spectrum can be broadened, and the likelihood of resistance development decreased or delayed (Gordillo Altamirano & Barr, 2021). The longevity of phage biocontrols will therefore be increased by utilizing diverse phages in cocktails, which will provide more consistent and robust control of bacterial infections.

**FIGURE 2:**
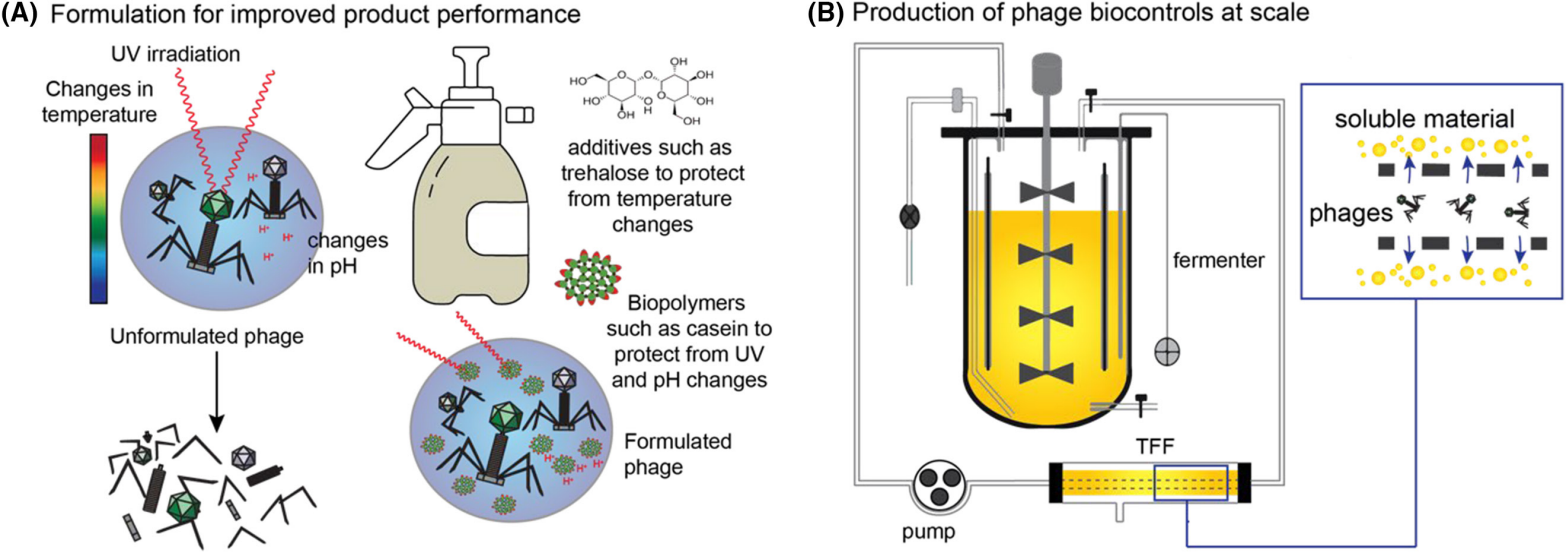
Methods to overcome issues phage biocontrols face. (A) Potential ways to formulate phages to increase their stability in application to crops for the control of bacterial pathogens to protect against phage degradation by UV, fluctuations in temperature and changes in pH. Additives such as trehalose and casein have been shown to increase phage stability *in planta* (Balogh et al., 2003; Malik et al., 2017). (B) Methods for the scale up of phage production for producing commercially available phage biocontrols. TFF, tangential flow filtration.

Despite the significant potential of phages for managing bacterial infections in crop production, several challenges must be overcome to enable the widespread adoption. These challenges include ensuring phage stability, obtaining regulatory approval, and achieving cost‐effective scalable production and distribution. Phage stability is a critical concern for effective application. Phages need to remain viable under various environmental conditions, including different temperatures, pH levels, and humidity, as well as exposure to ultraviolet light (Figure 2A). Formulations and protective carriers that can enhance phage stability during storage and application are key to the translation of in vitro results to the development of feasible products. Researchers are exploring ways to enhance phage delivery to the target bacteria (Figure 2A), such as encapsulating phages in protective polymers or combining them with other carriers that improve adherence to plant surfaces (Balogh et al., 2003; Malik et al., 2017). Additionally, recent research has shown that commensal plant microbes may show promise as delivery vectors of phage to diseased plants through fungal hyphae (You, Kallies, et al., 2022; You, Klose, et al., 2022). Formulation and novel delivery strategies would extend phage persistence and efficacy in the field. Integration into existing pest management programs is crucial for the success of phage therapy. The use of fermenters (García et al., 2019) and tangential flow filtration (Larsen et al., 2023) in the scale up of phage production have been explored (Figure 2B). However, thus far no standardized protocols for phage scale up in commercial application of phage biocontrols are publicly available.

Regulatory approval is another obstacle to the widespread adoption of phage therapy. The use of phages as biological control agents must meet stringent safety and efficacy standards to receive approval from regulatory bodies. The regulations that phage biocontrols are subject to will have regional dependencies and international products may face a greater number of hurdles than products developed domestically.

## ENDOLYSINS AS ANTI‐MICROBIAL AGENTS AGAINST PLANT PATHOGENS

Endolysins are lytic enzymes produced by phages at the end of their lytic lifecycle to cause cleavage of peptidoglycan and subsequent host lysis, resulting in the release of phage progeny. The exogenous application of endolysins as anti‐microbials against gram‐negative bacteria is hindered due to their inability to breach the impermeable outer membrane and reach the peptidoglycan (Sisson, Jackson, et al., 2024). However, they have been successfully applied against multiple gram‐positive bacteria. Our recent work has shown the potential of endolysins as anti‐microbials against *Psa* when used in combination with citric acid (Sisson, Fagerlund, et al., 2024). Chemical permeabilizers such as citric acid and ethylenediaminetetraacetic acid (EDTA) destabilize the outer membrane of gram‐negative species by chelating the divalent cations which bind to outer lipopolysaccharide (LPS) molecules. This results in an excess of electrostatic repulsion between LPS molecules and permeabilization of the outer membrane, allowing endolysins access to peptidoglycan. The environmental persistence of EDTA and its efficacy as an anti‐coagulant makes it an unattractive chemical for environmental application. Citric acid, however, is already commonly used pre‐harvest on kiwifruit to remove water spots (Eastpack, 2013), highlighting it as a potential synergistic partner for increasing endolysin efficacy in agricultural applications.

Additional methods to overcome the outer membranes of gram‐negatives for endolysin application include using either intrinsically active endolysins, or engineering endolysins by appending domains such as outer membrane penetrating peptides or receptor binding proteins (Sisson, Jackson, et al., 2024). However, at present, few native intrinsically active endolysins that target gram‐negative bacteria have been discovered and engineering of chimeric endolysins has been largely focussed towards human or zoonotic pathogens (Love et al., 2018). However, utilizing VersaTile DNA shuffling (Gerstmans et al., 2020) we have recently developed an endolysin fusion with exogenous activity against *Psa* (S. L. Warring, unpublished data). The development of such an endolysin illustrates the potential of engineering approaches for the development of endolysins targeted towards *Pseudomonas* pathovars.

## COMBINATION APPROACHES FOR THE IMPROVED CONTROL OF PHYTOPATHOGENS

While phages show promise as biocontrol agents against phytopathogens there are drawbacks and hurdles that must be overcome before they can be implemented as effective biocontrol agents. One such issue is the rapid development of bacterial resistance to phages. Indeed, our recent work has shown that *Psa* develops resistance to treatments with a single phage *in planta* and that resistance did not impair virulence of the pathogen (Warring et al., 2022). In this work, resistance was overcome by co‐evolving phages with phage resistant isolates, complementing our latest phage and host bacteria co‐evolution experiments utilizing phages targeting *Pss* (Rabiey et al., 2024). These co‐evolution experiments revealed that a powerful method to supress in vitro development of phage resistance was to use cocktails containing both wild‐type and coevolved phages. Both studies underscore the utility of sequencing analysis of phage resistant isolates and coevolved phages, and the power that genetic insight provides for the development of novel phage biocontrols.

In addition to phage cocktails, phages can be combined with known anti‐microbials such as antibiotics, chemicals, bacteriocins and endolysins to have synergistic effects and combat resistance (Greer et al., 2024; Mirski et al., 2019; Rendueles et al., 2022). Phage synergy with antibiotics has been recently illustrated for the zoonotic pathogens *Enterococcus faecium* and *faecalis* in synograms with phages and antibiotics including linezolid, ampicillin and vancomycin (Ghatbale et al., 2024). Data from this work showed that phages synergized with antibiotics to not only reduce MICs but also reversed previously developed antibiotic resistance. An important caveat of the study was that phage pressure needed to be maintained for the observed reversal of antibiotic resistance. Such work exemplifies the potential of combination approaches and should be extended to plant pathogens that affect various crops, with a goal to move away from antibiotics in food production to more sustainable alternatives. Since most pseudomonad infections are controlled in agriculture using copper and antibiotics, studies should look at potential synergy between phages and these chemical controls. However, caution should be used when considering phage synergy with copper as cupric ions have previously been shown to cause inactivation of some phages identified as biocontrol agents such as the ssRNA phage ϕ6 (Li & Dennehy, 2011; Molan et al., 2022) and the dsDNA *myoviridiae* phage ϕD5 (Czajkowski et al., 2017). Phage inactivation by copper appears to be dependent on phage (Li & Dennehy, 2011; Soliman et al., 2020), concentration (Czajkowski et al., 2017) and pH (Soliman et al., 2020). Therefore, studies should consider these variables when investigating interactions between phages and copper treatments. Other synergistic combinations that have been trialled thus far include phages with bacteriocins and bacteriocins with endolysins (Rendueles et al., 2022). Most combinations tested *in vitro* have shown high levels of synergy, coupled with a lack of resistance development. However, there is a paucity in the spectrum of pathogenic bacteria tested and the data is predominantly from *in vitro* studies. A key opportunity for future studies will be to investigate if the synergy demonstrated between bacteriocins and phages or endolysins translates effectively to *in planta* trials against phytopathogens. One currently untested combination is phages coupled with endolysins, providing another avenue for exploration for the development of novel and effective biocontrols.

## CONCLUSIONS

The devasting economic and societal impact of plant pathogens on crop production will worsen in the face of climate change and currently the tools used to combat these bacterial diseases are no longer fit for purpose. Phages present a desirable ecologically friendly alternative but there are still hurdles to be overcome before they can be effectively implemented at scale. An important obstacle that must be overcome is the development of resistance and limitations of host range for non‐clonal pathogens. It has been previously shown that while resistance does develop it can be overcome by co‐evolving phages with resistant strains and utilizing cocktails that target different host receptors. Additional tools to combat resistance are phage endolysins used either in concert with either organic acids or utilizing engineering approaches to overcome impermeable bacterial outer membranes for effective use against gram‐negative pathogens. Additionally, developing research interests point towards combined approaches where phage cocktails include antimicrobials such as antibiotics and/or bacteriocins for synergistic effects that prevent resistance development and reduce the amount of phage and anti‐microbial required. This research area has yet to be explored for plant pathogens and presents a promising pipeline for the development of biocontrols for use in crop production. By combining phage therapy with other control measures, growers can achieve more robust and sustainable management of bacterial infections in crop production.

## AUTHOR CONTRIBUTIONS

**Suzanne L. Warring:** Conceptualization; writing – original draft; writing – review and editing; funding acquisition. **Hazel M. Sisson:** Writing – review and editing. **Peter C. Fineran:** Writing – review and editing; conceptualization; funding acquisition. **Mojgan Rabiey:** Conceptualization; writing – original draft; writing – review and editing; funding acquisition.

## CONFLICT OF INTEREST STATEMENT

The authors declare that there are no conflict of interests.

## Data Availability

Data sharing is not applicable to this article as no new data were created or analyzed in this study.
